# COVID-19 cycles and rapidly evaluating lockdown strategies using spectral analysis

**DOI:** 10.1038/s41598-020-79092-6

**Published:** 2020-12-17

**Authors:** Guy P. Nason

**Affiliations:** Department of Mathematics, Imperial College, Huxley Building, 180 Queen’s Gate, London, SW7 2AZ UK

**Keywords:** Epidemiology, Statistics

## Abstract

Spectral analysis characterises oscillatory time series behaviours such as cycles, but accurate estimation requires reasonable numbers of observations. At the time of writing, COVID-19 time series for many countries are short: pre- and post-lockdown series are shorter still. Accurate estimation of potentially interesting cycles seems beyond reach with such short series. We solve the problem of obtaining accurate estimates from short series by using recent Bayesian spectral fusion methods. We show that transformed daily COVID-19 cases for many countries generally contain three cycles operating at wavelengths of around 2.7, 4.1 and 6.7 days (weekly) and that shorter wavelength cycles are suppressed after lockdown. The pre- and post-lockdown differences suggest that the weekly effect is at least partly due to non-epidemic factors. Unconstrained, new cases grow exponentially, but the internal cyclic structure causes periodic declines. This suggests that lockdown success might only be indicated by four or more daily falls. Spectral learning for epidemic time series contributes to the understanding of the epidemic process and can help evaluate interventions. Spectral fusion is a general technique that can fuse spectra recorded at different sampling rates, which can be applied to a wide range of time series from many disciplines.

## Introduction

During the UK Government COVID-19 briefing on 6th April 2020, the UK Deputy’s Chief Scientific adviser, Professor Angela McLean, said^1^ “We need a good long time series of data on all stages of infection in order to be able to tell what the impact of measures that came in on March 23 will be”. The measures that Professor McLean referred to were the widespread UK social distancing and lockdown interventions made in the face of the COVID-19 threat. At the time of writing, few countries have experienced in excess of 70 days of COVID-19 cases and most only have around 50 days. Professor McLean is correct in that many scientific inferences require longer time series than those currently available. However, we show that there are considerable and useful similarities in the underlying cyclic (spectral) behaviours of the numbers of new daily COVID-19 cases for a range of different countries (see "Appendix" figures). We use recent Bayesian spectral fusion methods^2^ (regspec) to pool spectral information across countries, which provides significantly more accurate estimates of cyclic behaviour than provided by a typical spectral analysis of a single country alone. The Bayesian principles underlying our fusion method mean that uncertainty is treated coherently, producing rational uncertainy assessment for our cycle (spectral) estimates. Our methods produce cycle estimates using the equivalent of over nine hundred daily observations, compared to the fifty or so that a typical standard spectral analysis might use. Using data^3^ from all of the countries we considered, our results show that transformed new daily COVID-19 cases have three underlying cycles: one operating at a wavelength of 2.7 days, a second at 4.1 days and a third at 6.7 days, which we take to be a weekly effect. We conducted separate analyses for the UK and groups of countries with similar spectra and note some variation in those cycles.

For some purposes it is not reasonable to compare or pool the number of new daily cases from one country to another^4^. For example, different countries might use different definitions of the number of daily cases and they record cases through different national structures and this is even the case for countries with political, geographical or cultural similarities. However, as long as the method of recording cases is broadly unchanged over the period in question for a particular country, the spectral properties across countries are comparable. The transformed cases’ spectrum quantifies the internal oscillatory structure within the series and, in terms of peak/trough identification, is largely unaffected by the overall level of cases, the different start times of epidemics in different countries (phase) and country-specific internal delays due to reporting requirements (also phase). In addition, the demonstration of the presence three consistent cycles across all countries, with some variation, provides supporting evidence for the suitability of the transformed new daily cases as a target of analysis, and comparisons between and across countries.

Another topic of great current interest is to ascertain whether and how a lockdown will influence the number of new daily COVID-19 cases. We consider this question for the group consisting of the UK, Italy, France, Germany, Spain, Switzerland, Belgium and the Netherlands. The number of days (with cases) before lockdown is, on average, 24.7 for this group of countries, and, after lockdown, is 23 (except the UK, which started its lockdown later). The averages just quoted allow for a five day incubation period. Our analysis compares the spectral properties before and after lockdown. A spectrum based on about 25 days worth of data would provide a very poor and highly uncertain estimate. However, our spectral fusion methods^2^ permit effective sample sizes for the group of 211 days worth of data prior to the lockdown, and 175 after, resulting in highly accurate spectral estimates for these periods. We learn that, after lockdown, the weekly cycle remains strong, but the shorter wavelength cycles become suppressed. This indicates that the weekly cycle is due, at least in part, to administrative recording effects, which are not effected by the lockdown.

The discovery of how the shorter wavelength cycles are disrupted by full lockdown suggests that they could be monitored during partial lockdowns. For example, if schools are reopened and the shorter wavelength cycles do not reappear, then this might indicate the effectiveness of that strategy. Given the similarity of the cycles across countries, this indicates that cases could be monitored and pooled across regions, over a short number of days to be fused into longer effective samples using the methods described here.

A considerably difficult problem is that of forecasting transformed new daily COVID-19 cases. Such information would be of great interest, e.g., to those planning health provision over a short timescale. Knowledge of the cycles is helpful, but we have had varying success in forecasting daily cases. However, with individual country series, with smaller number of days, it is unrealistic to expect too much and, in particular, the transformed cycles experience both a degree of time-modulation and possible frequency changes. More useful perhaps, are not daily forecasts, but the knowledge that the number of cases tend to increase and decrease over a period of three/four days. This means that if one observes a decrease in the number of daily COVID-19 cases after lockdown, that does not necessarily mean the peak has been reached, but is simply a manifestation of the 3/4 day cycles. Hence, one might believe a lockdown strategy has been successful after a sustained decrease of at least four days.

Spectral analysis^5,6^ of epidemics is not new, but most work has been carried out on epidemics observed over long time periods (seasons and years) using lengthy time series^7–9^. Recent work^10^ on COVID-19 has applied popular autoregressive integrated moving average process^5,11^models to a single prevalence time series with a sample size of $n=22$. However, conclusions derived from such analyses on a single series with such small sample sizes^12^ are questionable. For example, an autoregressive process of order one with parameter 0.9, normally considered to be a strong signal, is only distinguishable from white noise^13^ approximately 20% of the time with sample size of $n=22$; basic simulation studies show the large number of possible different models that can fit such short series apparently well. This indicates that it is virtually impossible to tie down the correct model with such a small sample size. Phenomenological sub-epidemic models^4,14^ show more promise and have been applied with some success to short-term forecasting of COVID-19 cases in Guangdong and Zhejiang, China. These improve performance by using bootstrap methods on short case time series, but are still ultimately based on a parametric model of single series. Our work is very different as it provides exceptionally accurate spectral estimates for a novel live epidemic that is still in its early days on short series, but reliably so by using recent Bayesian spectral fusion techniques^2^.The nonparametric nature of our analysis also permits us to split case time series at a boundary (e.g. lockdown or other intervention) and analyse the two halves separately, still with very short series in each. This is perhaps harder to do with classical parametric models and to maintain consistency between the two halves. On the other hand, our method relies on good quality case series from different regions, which are not always available for all epidemics.

## Results

### Transformed series, the UK spectrum and fusing the world and Europe

**Figure 1:**
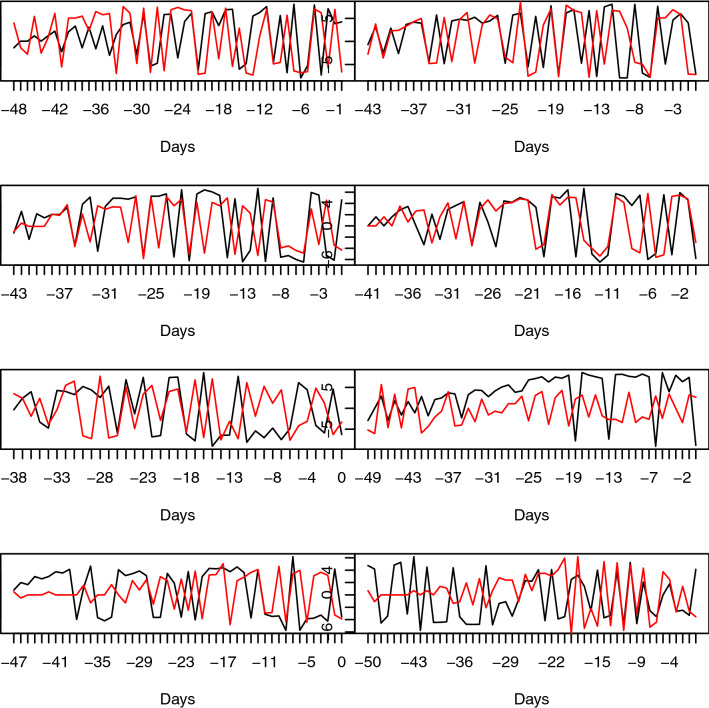
Number of daily cases on transformed scale for 16 countries. Left-to-right, top-to-bottom: UK, IT; FR, DE; ES, CH; BE, NL; AT, NO; US+CN; IR+CA; KR+AU. First country of pair in black, second is red.

We transformed the number of new daily COVID-19 cases by applying a signed log transform to the first differences of the new case time series (see Methods). The transformed number of new daily cases for 16 countries are shown in Fig. 1 each showing a distorted noisy, but characteristic sinusoidal trace.

**Figure 2:**
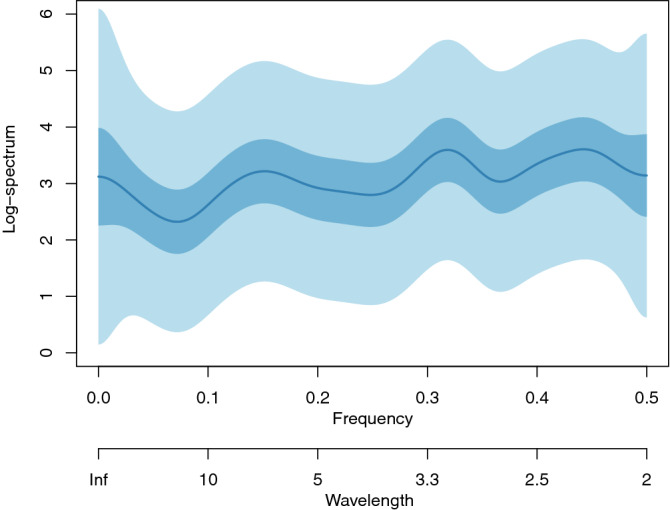
Bayesian log-spectral estimate of transformed UK new daily COVID-19 cases with 50% (dark blue shaded region) and 90% (light blue shaded region) credible intervals.

The estimated log-spectrum for the UK transformed new daily cases is shown in Fig. 2 and for all other countries we considered in the "Appendix" figures. Spectral estimates are commonly displayed on a logarithmic scale^15^. Spectral peaks can be observed in Fig. 2 at wavelengths of 6.7, 3.2 and 2.3 days, respectively. Although the peaks are visible, the credible intervals indicate that there is a fairly large degree of uncertainty, because this time series contains 52 observations. A frequentist analysis, e.g. using the spectrum function in R^15^, produces a similar result, but with even wider confidence bands.

Similar spectral analyses for each country indicate three similar spectral peaks, although not always as well-defined nor in precisely the same location.

**Figure 3:**
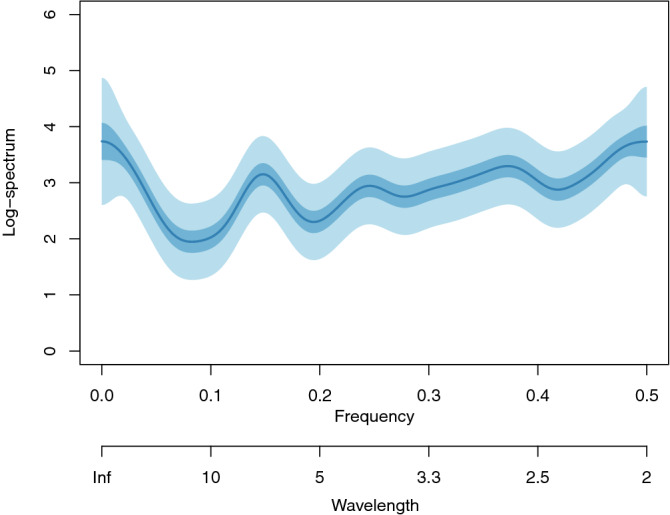
Bayesian log-spectral estimate of fusion of new daily COVID-19 cases for 18 countries with 50% (dark blue) and 90% (light blue) credible intervals. Countries included are the UK, Italy, France, Germany, Spain, Switzerland, Belgium, the Netherlands, Austria, Norway, the USA, China, Iran, Canada, South Korea, Australia, New Zealand and Sweden.

Figure 3 shows an estimate that is the result of coherently fusing spectra from 18 countries, giving an an effective sample size of 916 days. Here, the clear spectral peaks have narrow credible intervals, due to the large effective number of days afforded by using 18 countries together. The spectral peaks are located at wavelengths of 6.7, 4.1 and 2.7 days. The peak around 6.7 days is observed in the spectral plots for individual countries and we interpret it to be a weekly effect. Such a weekly effect could be produced by reporting artefacts (e.g. paperwork being delayed until Monday, or carried out differently at the weekend) or due to the behaviour differences of people at weekends. All countries analysed have a 5+2 working week/weekend pattern, although not necessarily the same days of the week (the particular specific weekend days amount to a phase effect, which does not affect the spectrum).

### Clustering spectra and groups of countries with similar spectra

**Figure 4:**
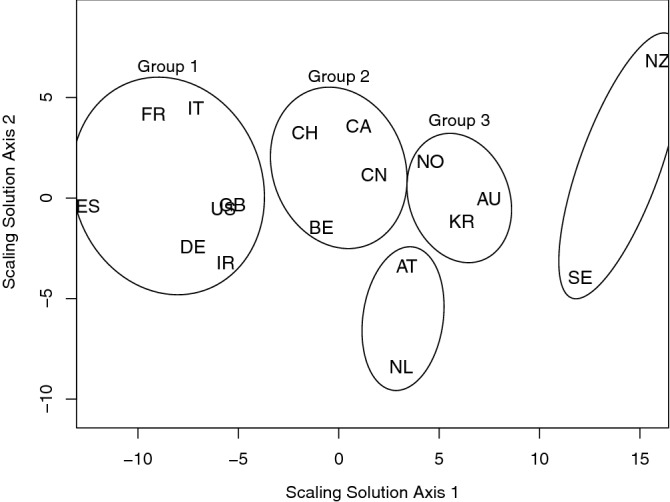
Multidimensional scaling solution of dissimilarity matrix generated by Euclidean distances calculated between two spectral estimates for each pair of countries. Countries’ position denoted by their two character ISO 3166-1 standard abbreviation. The ellipses group together countries that are clustered using the robust cluster stability method^16^. AT = Austria, AU = Australia, BE = Belgium, CA = Canada, CH = Switzerland, CN = China, DE = Germany, ES = Spain, FR = France, GB = United Kingdom, IR = Iran, IT = Italy, KR = South Korea, NL = Netherlands, NZ = New Zealand, NO = Norway, SE = Sweden, US = USA.

We next clustered our 18 countries based on their spectrum, by calculating a dissimilarity between the spectra for each pair of countries, and then performing both a hierarchical cluster analysis and multidimensional scaling on the dissimilarity matrix. The scaling solution indicated that only two dimensions were required to encapsulate 72% of variation in the data. Figure 4 shows the resultant two-dimensional solution.

Attaching a precise meaning to the scaling axes in Fig. 4 is not easy in that the origin and orientation of the presented configuration is arbitrary in this method^17^. What counts is the relative position of the countries to each other, which are arranged according to the spectral distance between each pair of countries, which is in units equivalent to the (local) log-variance of the series. We speculate that Axis 1 might indicate how badly a country has been perceived to have been affected by the virus with Australia, New Zealand and Sweden less so and those on the left of the plot considerably more so. However, Germany is the obvious anomaly to this interpretation as, currently, it has perhaps been perceived to have handled the crisis well so far.

**Figure 5:**
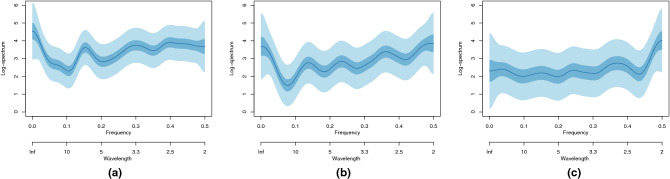
Bayesian log-spectral estimates and 50% and 90% credible intervals for (**a**) Group 1 countries: Spain, France, Italy, the US, the UK and Iran. Effective number of days=357; (**b**) Group 2 countries: Switzerland, Canada, Belgium and China. Effective number of days=229; (**c**) Group 3 countries: Norway, Australia and South Korea. Effective number of days = 157.

Figure 5 shows the spectral estimates for each of the three groups of countries identified in Fig. 4, using the clustering techniques described in Methods.

The peak frequencies for each of these groups is listed in Table 1, which shows differences between them. However, each group possesses a possible weekly peak and higher-frequency peaks labelled a., of around three to four days, and b., around 2.6 days. The Group 3 fused spectrum looks qualitatively quite different to Groups 1 and 2 in that Group 3 does not appear to have any strong seven day cycle, the shorter wavelength peaks are flatter and most power appears around the highest frequencies.

**Table 1:** Spectral peaks for the three country groups in units of days. The peaks in the second and third rows have been arbitrarily labelled as peak (a) and (b). The peak values have been taken by manual location and, of course, are subject to error.

Peak	Group 1	Group 2	Group 3
Weekly	6.48	7.27	6.59
a.	3.31	4.30	4.09
b.	2.52	2.77	2.70

### Spectral changes after lockdown

Many countries experiencing the COVID-19 pandemic instituted a range of lockdown measures to dramatically reduce virus spread. At the time of writing, these countries have observed new daily COVID-19 cases for between 43 and 54 days. We assume that, on average, it takes about five days for the virus to incubate. Table 2 shows the start and end date for each time series used in the pre- and post-lockdown spectral estimates to follow. It also shows the date we used for transition between pre- and post-lockdown periods and the total number of observations in the case series along with the numbers before and after the transition date.

**Table 2:** Start and end dates and number of observations of cases time series. The transition date used, including incubation time of five days, and the number of observations used in the ‘pre’ and ‘post’ lockdown period.

Country	Time series dates			Number Obs.		
	Start	‘Transition’	End	Total	Before	After
United Kingdom	22 Feb	28 Mar	13 Apr	52	38	14
Italy	20 Feb	14 Mar	13 Apr	52	24	28
France	24 Feb	18 Mar	12 Apr	49	26	23
Germany	24 Feb	22 Mar	12 Apr	49	30	19
Spain	24 Feb	16 Mar	12 Apr	49	24	25
Switzerland	26 Feb	18 Mar	12 Apr	47	24	23
Belgium	1 Mar	19 Mar	12 Apr	43	21	22
Netherlands	28 Feb	20 Mar	12 Apr	45	24	21
Total				386	211	175

A precise, single, lockdown date is not easy to discern for every country. For the UK it is generally assumed that the virus was spreading more or less evenly across the country and the whole country was instructed to lockdown on one date by the Prime Minister in televised announcement on 23rd March. Hence, adding on the five days assumed incubation period gives us the transition date of 28th March as shown in Table 2. For other countries, e.g. Switzerland, lockdown was a more prolonged process, restricting different activities in the country over a series of days. As another example, Italy enacted regional lockdowns before enacting more stringent national restrictions. We chose the transition dates in Table 2 by reference to the various lockdown actions reported in each country^18^ and by examining the percentage change in retail and recreation activity from the Google Mobility Reports for each country^19^. The Mobility Reports show that activity for all countries started to decline in advance of the official lockdown date (where a single date could be identified) and continued afterwards. Hence, we select a ‘lockdown’ date that is roughly halfway during the main period of decline, consistently for each country

**Figure 6:**
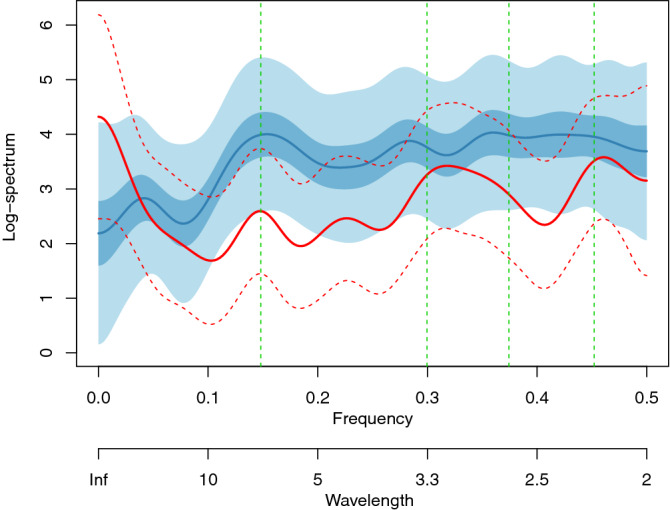
Pre-lockdown Bayesian log-spectral estimate (red solid line) and 90% credible interval (red dashed lines). Post-lockdown log-spectral estimate (blue solid line) and 50% and 90% credible intervals. Green vertical dashed lines from left to right indicate wavelengths of 6.74 (weekly), 4.42, 3.14, and 2.17 days.

The number of days before and after the lockdown are, in each case, too small to carry out anything other than the most simplistic time series to maintain statistical reliability for individual countries. In particular, a spectral estimate in this situation would be subject to a high degree of uncertainty. However, Fig. 6 shows our coherently fused spectral estimates^2^ across these countries before and after the lockdown period, making use of 211 effective days prior to lockdown and 175 days afterwards.

The weekly peak is clearly visible in both estimates. There are pre-lockdown peaks at wavelengths of 4.42, 3.14 and 2.17 days. After lockdown all of these peaks are suppressed relative to the rest of the spectrum and, indeed, the first two have turned into troughs. This result is particularly interesting as it suggests that the non-weekly peaks have been severely disrupted by the lockdown. The weekly effect seems relatively unchanged by the lockdown, indicating that it was driven by non-epidemic effects, such as recording/paperwork or bureaucracy caused by weekends.

The post-lockdown spectrum is higher overall than the pre-lockdown spectrum, this is due to the larger variation associated with the larger number of cases identified during the progress of the epidemic. Our transformation suppresses this variation, but does not remove it entirely.

There is considerable uncertainty around the value of the incubation period for COVID-19. The current consensus appears to be around five days^20^, which Fig. 6 uses. Recent literature reports a range of incubation periods such as a medians of four days (interquartile range of two to seven)^21^, of 5.1 days (with 95% confidence interval of 4.5 to 5.8 days)^22^ or of 6.06 days (with 95% confidence interval of 5.84 to 6.29 days)^23^. So we repeated the analysis that led to Fig. 6 and a broadly similar picture emerges if we change the incubation period to three, four, seven, eight and nine days, but is inconclusive for six days.

## Discussion

Early on within an epidemic various characteristic time series (cases, deaths, hospitalizations) tend to be short and it is unreasonable to expect them to provide accurate information on quantities of interest, such as flexible trend and stochastic structure such as autocovariance and spectral quantities. When examining the effect of pre- and post-interventions, the length of the component series can be shorter still. Our work uniquely provides a way for coherently fusing spectral information using a Bayesian method to provide accurate spectral estimates that can provide information such as cycles of varying wavelength as exemplified here. Such cycle knowledge provides useful information such as that to explain sudden drops in numbers of (e.g.) cases, which are observed early on by experts and the media alike as evidence for epidemic control, but really were just manifestations of the intrinsic cycle. Knowledge of cycles also indicates the approximate length of time that cases (e.g.) need to be observed before genuine rises or falls can be believed.

The analysis above reveals strong weekly cycles (at wavelengths just short of seven days) and other cycles typically operating around 2.7 and 4.1 days. Other individual countries and mixtures of countries in fused estimates do occasionally give rise to other cycles or cycles with wavelengths slightly shifted from those quoted, but the approximate agreement across several countries is remarkable. Such similarities provide justification for the spectral fusion method itself, but also the claim that epidemic response in countries with different measuring criteria and systems can be compared in terms of spectral quantities. This is because a spectral quantity is a relatively defined quantity and assessing the frequency behaviour of a time series, irrespective of the measurement units or system used. Hence, it then is possible to group countries using cluster analysis applied to the spectral estimates as discussed above where Spain, France, Italy, the USA, the UK, Germany and Iran formed a single group (Group 1), Switzerland, Canada, China and Belgium formed Group 2, Norway, Australia and South Korea formed Group 3, Austria and the Netherlands and, separately, New Zealand and Sweden formed a final group (although the latter two are probably better understood as not being particularly similar, but just not members of Groups 1–3.

We analysed numbers of deaths using similar methods described here and found similar cycles. Although we have not carried out a detailed analysis, if the number of deaths process can be approximated by a linear system^5,11^ with the numbers of cases as input, then similar cycles are to be expected.

A time series with a fixed sampling rate and length has a minimum and maximum (Nyquist) frequency range that can be observed^5,11^. Although our spectral fusion methods^2^ provide more accurate estimates of the spectrum in that range (equivalent to having a larger sample size), they can not provide information on frequencies outside of it. To estimate lower frequencies, we would need a genuinely longer series and, for higher frequencies, we would require cases more frequently than once a day, which do not seem to be routinely collected by official bodies. However, if a country decided to release case numbers on some other sampling plan (e.g. every two days, or weekly) then regspec would be able to fuse the spectral estimates. Such a feature might be of use when dealing with reporting structures that are not equipped to provide daily reporting of cases or where weekly cases are thought to be more accurate. For example, this might apply to regions with fragile health or reporting systems or populations that are spread across widely dispersed geographical regions with poor communications.

Our analyses assume approximate stationarity and linearity for the transformed series, which is unlikely to be exactly true in practice. For example, in the UK transformed case series, there are hints of the series oscillation speeding up over the last ten days. Practically speaking, changes in the testing regime, recording practices, the lockdowns or other interventions will change the dynamics of either the pandemic itself or recording of it. Ideally, it would be of interest to use methods for non-stationary time series^24,25^, but the current series available to us are far too short for such analyses.

We also attempted some short-term forecasting of the COVID cases and deaths series with our short time series and results were not particularly good. We used autoregressive moving average (ARMA)^11^ models and direct least-squares fitting of linear combinations of sinusoids and, as is often the case with time series, especially short ones, the model fit was acceptable, but forecast results were very poor. For these short time series it is probably the case that multiple models in the rich ARMA class (for example) fit well, but are not really reflective of the true nature of the time series. For some time series models it can be that hundreds of observations need to be collected before a unique correct model can be reliably ascertained^13^. Secondly, it seems likely that these series will not be stationary (in first or second order), but they might be locally or piecewise stationary, so forecasting methods might only work well for some stable periods.

The spectral peaks are not always well-defined, nor precisely in the same location and, indeed, occasionally more than three peaks appear, such as in the pre-lockdown estimate shown in Fig. 6. The Bayesian credible intervals in our figures permit us to coherently quantify our uncertainty around these peaks. As well as the clear weekly signal, the repeated appearance of similar wavelengths in multiple plots for multiple regions lends credibility to their actuality. From Table 1 it should be noticed that the ratio $6.48/3.31 \approx 1.96 \approx 2$ exactly, so the (a.) peak might be a harmonic of the weekly signal. However, the (b.) peak does not appear to be a harmonic.

A further intriguing possibility would be to examine the spectra of short series arising from regions within a country. Some countries do possess a regional reporting system that adheres to a robust national data standard, for example Brazil or the USA. In this case, the spectral fusion might be even more useful for short series as, presumably, there will be fewer extraneous factors that distort spectra on a regional basis.

## Methods

All computations were executed in R^15^ and packages that are mentioned specifically below.

### COVID-19 new daily cases transformation

Let $Y_t$, for $t=1, \ldots , n_c$ represent the number of new daily cases for $n_c$ days for country *c*. The spectral dynamics of the number of daily cases for different countries are all countries masked by the well-known and characteristic exponential increases (and decrease, for those countries that locked down and have now passed their peak). Hence, we transform our number of daily cases series to reveal the spectral dynamics. After exploratory data analysis^11^ the following transform was used for all series $L_t = {\text {sgn}}(D_t) \log ( | D_t |)$, where the sign function ${\text {sgn}}(x)$ is $+1$, if *x* is positive or $-1$, if *x* is negative, and $D_{t} = Y_{t } - Y_{t-1}$ for $t=2, \ldots , n_c$. The transform is easily inverted (if $Y_1$ is retained).

### Bayesian spectral estimation and fusion: Regspec

We use the regspec^2,26^ Bayesian spectral estimation method with a neutral white noise prior with prior variance of 1 and all default arguments, except for a smoothing parameter of 0.7, although the results are not sensitive to the latter. Regspec straightforwardly enables the production of spectral estimates using multiple data sets, with each having different lengths and produces coherent credible intervals to properly ascertain the uncertainty inherent in the estimation process. Regspec can also fuse spectra for multiple series recorded at different sampling rates, but we do not need to use this feature here as all our time series are recorded daily.

### Clustering of spectra

Although the number of cases transformed time series show similar spectral behaviour they are not identical. However, it is possible to observe closer similarities within certain subgroups of countries. We used unsupervised clustering and scaling techniques^17,27^ to depict the relationship between different countries and suggest a clustering for them. First, for each country we produced a spectral estimate using regspec as mentioned above, and then formed a dissimilarity for each pair of countries by computing the Euclidean distance between their log-spectral values (using the dist function in R^15^). Classical multidimensional scaling was then used to produce an estimated two-dimensional configuration using the cmdscale function in R^15^. For clustering we use hierarchical cluster analysis on the dissimilarity matrix. It is well-known that dendrograms are sensitive to the input dissimilarity matrix, so we used the clusterwise cluster stability assessment by resampling method to produce a stable clustering^16^. We use the R hclust algorithm^28^ and the ward.D2 agglomeration method^29,30^.

## Figures and Tables

**Figure 7:**
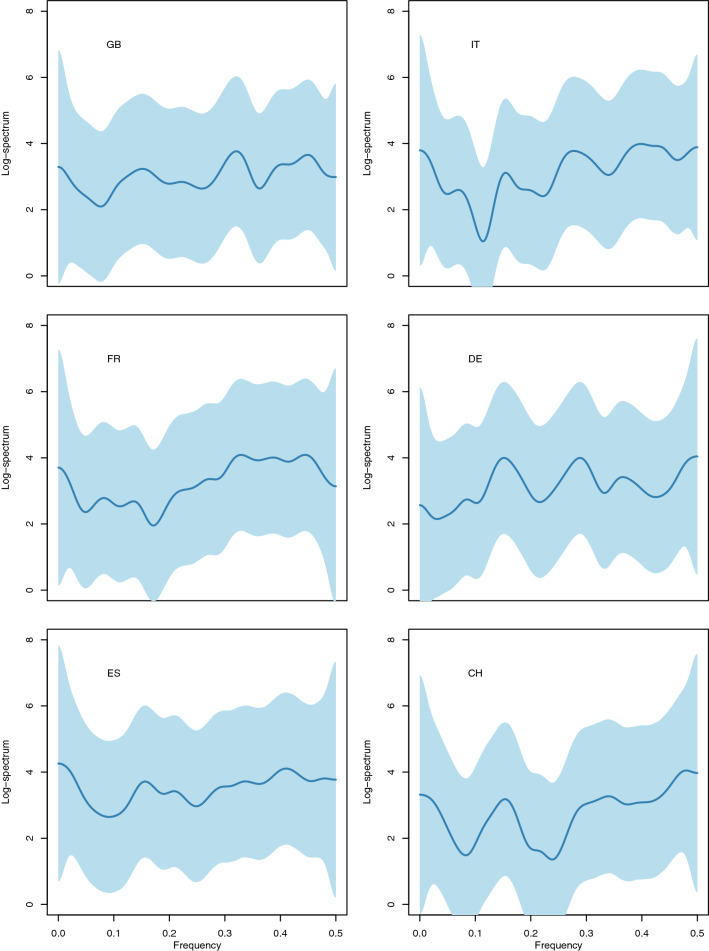
Regspec spectral estimates for numbers of new daily COVID-19 cases. Top-to-bottom, left-to-right: United Kingdom on 52 days, Italy on 54 days, France on 49 days, Germany on 49 days, Spain on 49 days, Switzerland on 47 days.

**Figure 8:**
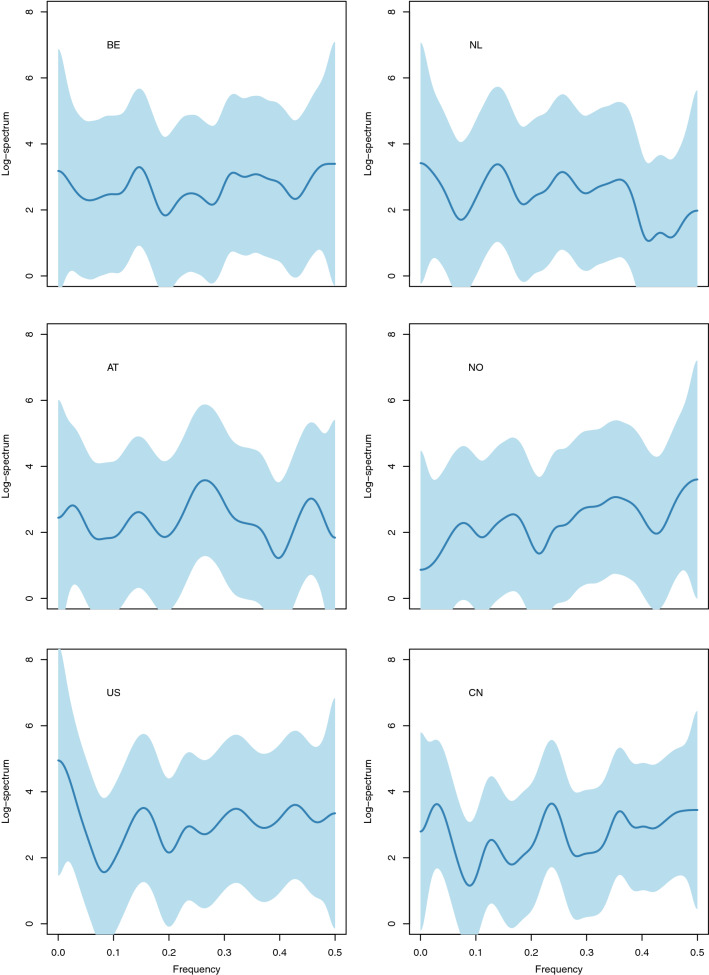
Regspec spectral estimates for numbers of new daily COVID-19 cases. Top-to-bottom, left-to-right: Belgium on 43 days, the Netherlands on 45 days, Austria on 49 days, Norway on 47 days, the USA on 53 days, China on 87 days.

**Figure 9:**
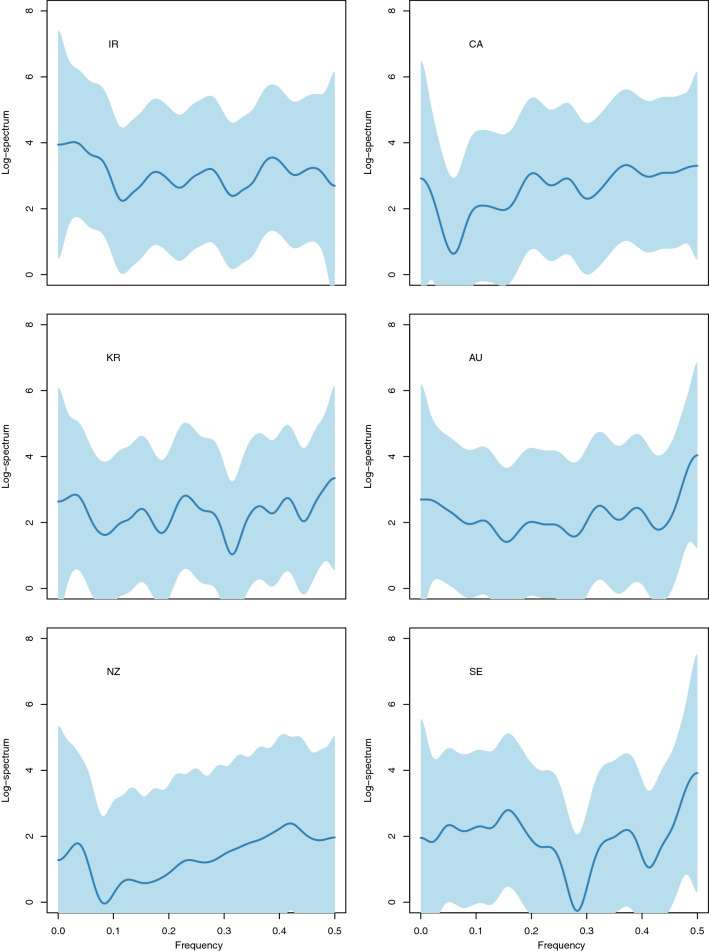
Regspec spectral estimates for numbers of new daily COVID-19 cases. Top-to-bottom, left-to-right: Iran on 55 days, Canada on 49 days, South Korea on 56 days, Australia on 54 days, the New Zealand on 28 days, Sweden on 47 days.

## Data Availability

The number of daily COVID-19 cases for countries can be found at the website of the European Centre for Disease Prevention and Control^3^.

## Appendix

Extended Data Figs. 7, 8, 9 are displayed on the next pages. Start and end dates for series in the lockdown comparison was given in Table 2. For the remaining countries the end date was always 12th April and the length of series and start dates as shown in Table 3.

**Table 3:** Start date and number of observations of cases time series.

Country	Start	No. Days	Country	Start	No. Days
Austria	24th Feb	40	New Zealand	14th Mar	26
Australia	19th Feb	52	Norway	26th Feb	45
Canada	24th Feb	49	South Korea	17th Feb	54
China	17th Jan	87	Sweden	26th Feb	47
Iran	18th Feb	53	USA	20th Feb	51

## Code availability

Full code to reproduce our methods and figures can be found in the zenodo archive with DOI is 10.5281/zenodo.4081366.

## References

[1] Drewett, Z. We don’t know if coronavirus deaths will peak this week. https://metro.co.uk/2020/04/06/dont-know-coronavirus-deaths-will-peak-week-12517835/ (2020).

[2] Nason GP, Powell B, Elliott D, Smith P (2017). Should we sample a time series more frequently? Decision support via multirate spectrum estimation (with discussion). J. R. Stat. Soc. A.

[3] European Centre for Disease Prevention and Control. Covid-19 cases worldwide. https://www.ecdc.europa.eu/en/publications-data/download-todays-data-geographic-distribution-covid-19-cases-worldwide (2020).

[4] Roosa K (2020). Short-term forecasts of the COVID-19 epidemic in Guangdong and Zhejiang, China: February 13–23, 2020. J. Clin. Med..

[5] Priestley MB (1983). Spectral Analysis and Time Series.

[6] Percival DB, Walden AT (2010). Spectral Analysis for Physical Applications.

[7] Grenfell B, Bjørnstad O, Kappey J (2001). Travelling waves and spatial hierarchies in measles epidemics. Nature.

[8] Conlan A, Grenfell B (2007). Seasonality and the persistence and invasion of measles. Proc. R. Soc. Ser. B.

[9] Ferrari M (2008). The dynamics of measles in sub-Saharan Africa. Nature.

[10] Benvenuto D, Giovanetti M, Vassallo L, Angeletti S, Ciccozzi M (2020). Application of the ARIMA model on the COVID-19 epidemic dataset. Data Brief.

[11] Chatfield C (2003). The Analysis of Time Series: An Introduction.

[12] Hyndman R, Athanasopoulos G (2013). Forecasting: Principles and Practice.

[13] Nason G, Savchev D (2014). White noise testing using wavelets. Stat.

[14] Chowell G, Tariq A, Hyman J (2019). A novel sub-epidemic modeling framework for short-term forecasting epidemic waves. BMC Med..

[15] R Development Core Team (2009). R: A Language and Environment for Statistical Computing.

[16] Hennig, C. *fpc: Flexible Procedures for Clustering* (2020). R package version 2.2-5.

[17] Chatfield C, Collins A (1980). Introduction to Multivariate Analysis.

[18] Wikipedia. Covid-19 pandemic in europe. https://en.wikipedia.org/wiki/COVID-19_pandemic_in_Europe (2020).

[19] Google. Covid-19 community mobility reports. http://www.google.com/covid19/mobility/ (2020).

[20] Wikipedia. Covid-19 pandemic. https://en.wikipedia.org/wiki/COVID-19_pandemic (2020).

[21] Guan W-J (2020). Clinical characteristics of coronavirus disease 2019 in China. N. Engl. J. Med..

[22] Lauer S (2020). The incubation period of coronavirus disease 2019 (COVID-19) from publicly reported confirmed cases: estimation and application. Ann. Intern. Med..

[23] Fang L-Q (2020). Meteorological conditions and nonpharmaceutical interventions jointly determined local transmissibility of COVID-19 in 41 Chinese cities: A retrospective observational study. Lancet Reg. Health West. Pac..

[24] Dahlhaus R, Subba Rao T, Subba Rao S, Rao C (2012). Locally stationary processes. Handbook of Statistics.

[25] Nason GP (2013). A test for second-order stationarity and approximate confidence intervals for localized autocovariances for locally stationary time series. J. R. Stat. Soc. B.

[26] Powell, B., Nason, G. P., Elliott, D. & Smith, P. *regspec: Non-parametric Bayesian spectrum estimation for multirate data* (2016). R package version 2.4.

[27] Hastie T, Tibshirani R, Friedman J (2009). The Elements of Statistical Learning.

[28] Murtagh F (1985). Multidimensional Clustering Algorithms.

[29] Ward J (1963). Hierarchical grouping to optimize an objective function. J. Am. Stat. Assoc..

[30] Murtagh F, Legendre P (2014). Ward’s hierarchical agglomerative clustering method: which algorithms implement Ward’s criterion?. J. Classif..

